# Decomposing Inequality in Long-Term Care Need Among Older Adults with Chronic Diseases in China: A Life Course Perspective

**DOI:** 10.3390/ijerph17072559

**Published:** 2020-04-08

**Authors:** Han Hu, Yafei Si, Bingqin Li

**Affiliations:** 1 School of Public Policy and Administration, Xi’an Jiaotong University, No. 28 Xianning West Road, Xi’an 710049, Shaanxi, China; huhan112@xjtu.edu.cn; 2 ARC Centre of Excellence in Population Ageing Research (CEPAR), The University of New South Wales, Sydney, NSW 2052, Australia; 3 Social Policy Research Centre, The University of New South Wales, Sydney, NSW 2052, Australia; bingqin.li@unsw.edu.au; 4 Centre for Social Development in Africa, University of Johannesburg, Johannesburg, Auckland Park 2006, South Africa

**Keywords:** long-term care need, ADLs, IADLs, inequality, concentration index

## Abstract

*Background*: China has the largest number of aging people in need of long-term care, among whom 70% have chronic diseases. For policy planners, it is necessary to understand the different levels of needs of long-term care and provide long-term care insurance to ensure the long-term care needs of all people can be met. *Methods*: This study combines the 2013 wave of CHARLS survey and the Life Course Survey of 2014. The combination allows us to factor in both childhood and adulthood data to provide life-course analysis. We identified 7,734 older adults with chronic diseases for analysis. The need for long-term care is defined by the presence of functional limitations based on the performance of basic activities of daily living (ADLs) and of instrumental activities of daily living (IADLs). Two dummy variables, ADLs disability and IADLs disability, and two count variables, ADLs score and IADLs score, were defined to measure incidence and severity of long-term care need, respectively. The concentration index was used to capture the inequality in long-term care need, and a decomposition method based on Probit Regression and Negative Binomial Regression was exploited to identify the contribution of each determination. *Results*: At least a little difficulty was reported in ADLs and IADLs in 20.44% and 19.25% of respondents, respectively. The concentration index of ADLs disability, ADLs score, IADLs disability, IADLs score were −0.085, −0.109, −0.095 and −0.120, respectively, all of which were statistically significant, indicating the pro-poor inequality in the incidence and severity of long-term care need. Decomposition analyses revealed that family income, education attainment, aging, and childhood experience played a significant role in explaining the inequalities. *Conclusions*: The long-term care need among older adults with chronic disease is high in China and low socioeconomic groups had a higher probability of needing long-term care or need more long-term care. It is urgent to implement long-term care insurance, especially for the individuals from lower socioeconomic groups.

## 1. Introduction

According to the National Bureau of Statistics of China, 249 million people were over 60 or older in China at the end of 2019, accounting for 17.9% of the total population [1]. The proportion of old population is projected to increase to 27.8% of the total population by 2040. Of old adults, 18.3% was in need of long-term care and that proportion was also estimated to increase significantly in the following decades [2]. The long-term care includes informal care provided by family members and formal care provided by institutions. The latter can be offered at older people’s home or in care homes. Because of the One Child Policy and population aging, the dependent ratio in China is increasing, which means the capacity of families to provide informal care is declining [3,4]. Institutional formal care in China is also insufficient to address the needs of frail older and disabled population, resulting in salient unmet need of long-term care. Among these older people, there are also great disparities in the need for care and the ability to pay for it [5]. How to address the need of long-term care is a growing social concern in China and there is urgent need of evidence for policy making. In 2016, the Chinese government launched a long-term care insurance in 15 pilot cities to provide affordable and accessible basic life care and daily nursing services for the disabled and elderly [6]. 

Developing sustainable and equitable long-term care system is the main objective set by the World Health Organization. Therefore, one important agenda often discussed is whether and how to extend the pilot of long-term care insurance to universal coverage in China. It is reasonable to seek clarification of one key question, the distribution of long-term care needs, before the expansion. Old people need long-term care services when their physical function is deteriorated or limited, especially the ability to perform the most basic and common activities [7,8]. Increasing evidence supports the notion that physical health in the old-age period can be affected not only by the health of individuals through the aging process [8], but also through the cumulative effect of socio-economic disadvantages during childhood [9]. It is well documented that insufficient income or low socioeconomic status in old age can be a predictor of low status of physical health, underutilization of health care, and mental diseases [3,7,10]. These are mostly findings in developed countries, while there are few researches on low-and-middle income countries on this subject. In addition, childhood may represent a key life stage for designing interventions to address health inequality in later life and socioeconomic status in childhood stage also influences the incidence and progression of physical, cognitive, and mental health in old life [9,11,12]. Therefore, the value of life course approach or perspective has been increasingly recognized in the study of aging worldwide [12].

This study aims to investigate the income-related inequality in long-term care need among older adults with chronic diseases in China through a life course perspective. The first rationale is that chronic diseases exerted high demand for healthcare and have a strongly positive association with physical disability of older adults [6,13,14]. Thus, the characters of this group’s need of long-term care and its distribution will be one key part to consider establishing a universal coverage of long-term care insurance in China. Second, we focus on the specific group of population for the potential crowding-out effect of long-term care need on healthcare utilization. Without long-term care insurance, the out-of-pocket payment for long-term care services is much more expensive or even unaffordable. Thus in China, individuals with long-term care needs may choose to utilize healthcare services, especially inpatient care, because they can be subsidized by health insurance [6,15,16]. Furthermore, we decompose the inequality based on regression model and contribute to the literature with the role of adulthood socioeconomic status in a short-term and the role of childhood experience in a long term on the need of long-term care. Accordingly, a better understanding of the inequality in long-term care need will help policymakers with the identification of older adults in the severe need of long-term care.

## 2. Methods

### 2.1. Data Source

To include factors both from childhood and adulthood, this study used two waves of the national representative data of the China Health and Retirement Longitudinal Study (CHARLS), including the 2013 wave of CHARLS and the 2014 life course survey [17]. The two datasets follow the same group of interviewees. A unique individual ID is used to combine the two datasets. More details about the data can be accessed on the website (http://charls.pku.edu.cn/en). In this study, given physician diagnosed condition including hypertension, dyslipidemia, diabetes etc., a subsample of respondents with chronic diseases were selected. After cleaning data with important information missing including ADLs/IADLs, family income, chronic diseases, gender, age as well as childhood experience, we have 7734 respondents identified for analysis. In this study individual weights were used to adjusted for non-response to obtain robust results.

### 2.2. Ethics Approval

Ethics approval for the study was granted by the Ethics Review Committee of Peking University, and all the participants provided signed informed consent at the time of participation. The study methodology was carried out in accordance with approved guidelines. 

### 2.3. Outcomes

Long-term care need is measured by the presence of functional limitations or disability [5,7,18]. The most common approach is based on the performance of basic activities of daily living (ADLs) and instrumental activities of daily living (IADLs) [7,18]. ADLs includes dressing, bathing, toileting, eating, getting in/out of bed, and controlling urination and defecation. IADLs includes as household chores, shopping, and taking medicine. 

In this study, four indicators, ADLs disability, IADLs disability, ADLs score, and IADLs score, were defined to measure the incidence and severity of long-term care need based on the performance of basic activities of daily living (ADLs) and instrumental activities of daily living (IADLs). ADLs disability and IADLs disability were two dummy variables to measure incidence of the need (1-Yes, 0-No). They were coded as 1 when the individual reported having difficulties in at least one dimension of ADLs/ IADLs and coded as 0 when no difficulties reported. ADLs score, and IADLs score were two count variables aiming to measure the severity of long-term care need. For each dimension of ADLs/IADLs, the score ranges from 0 to 3. It was scored 0 when no difficulty reported, 1 when little difficulty reported, 2 when much difficulty reported, and 3 when the individual without any ability to do it. Therefore, ADLs score ranges from 0 to 18 while IADLs score ranges from 0 to 12. A higher score indicates more difficulty in keeping daily activities well and more needs of long-term care.

### 2.4. Exposure of Interest

In this study, we focused on the income-related inequality in long-term care need among adults with chronic diseases, therefore the most important independent variable of interest was economic status. It was measured by annual family consumption per-capita. It was supported by recent research efforts that family consumption is better than family income when quantifying economic status [15,16]. Furthermore, the annual family consumption per-capita was divided into five groups according to their ranking, i.e., Quantile_1 (the poorest group), Quantile_2 (the poorer group), Quantile_3 (the middle group), Quantile_4 (the richer group) and Quantile_5 (the richest group). We also performed robust check by using logarithm of annual family consumption per-capita. 

### 2.5. Other Covariates

Besides, a series of independent variables included in CHARLS dataset were taken into account based on a comprehensive literature review [3,5,8,19,20,21,22,23,24,25,26]. Demographic characteristics included gender (0-Female and 1-Male), age group (0-Age 45–54, 1-Age 55–64, 2-Age 65–74 and 3-Age ≥ 75), household size (0-members 1–2, 1-members 3–4, 2-members 5–6, 3-members 7 and above) and ethnicity (0-Minority and 1-Han ethics). Adulthood socio-economic characteristics included highest educational attainments (0-Illiteracy, 1-Primary school, 2-Middle school and 3-High school), health insurance coverage (0-Non-coverage, 1-Basic health insurance and 2-Business health insurance), Hukou registration (0-Agriculture and 1-Non-agriculture), living in urban (0-No and1-Yes), and communist party member (0-No and1-Yes). Adulthood health behavior characteristics included alcohol consumption (0-No and1-Yes) and tobacco consumption (0-No and1-Yes). 

A set of variables were included to identify the long-term effect of childhood environment and health. CHARLS included a rich set of questions acquiring information about childhood experiences. Hunger experience was defined by question “At what age ranges did this (your family had no enough food to eat) happen?” It was reported with three periods, age 0–5, age 6–12, and age 13–17 (0-None of the three periods, 1-Period 1, any one of the three periods, 2-Period 2, any two of the three periods and 3-Period 3, all of the three periods). Receiving any vaccinations under 15 (0-No and1-Yes) was derived from the question “Before you were 15 years old (including 15 years old), have you received any vaccinations?” According to the question “Have you always had a usual source of care, that is, a particular person or a place that you went to when you were sick or you needed advice about your health?”, we defined the variable of daily medical source. Child health was defined following the question “Before you were 15 years old (including 15 years old), would you say that compared to other children of the same age, you were 15 (0-Much healthier, 1-Somewhat healthier, 2-Average, 3-Less healthy and 4-Much less healthy)”. Finally, province fixed effect was controlled in the regression model to exclude geographic variation [26]. 

### 2.6. Statistical Analysis

The inequality in long-term care need was estimated by using the concentration index [27], which was further decomposed into its determinants based on regression models [15,16,28,29,30]. Probit Regression model was used for dummy variables and Negative Binomial Regression model was used for count variables to estimate the partial effect of each covariate (Equation (1)):

(1) $$y=\alpha^{m}+\sum_{i}\beta_{i}^{m}x_{i}+\sum_{j}\beta_{j}^{m}x_{j}+\varepsilon$$

where y is long-term care need (ADL disability, IADL disability, ADL score, and IADL score), $x_{i}$ is adulthood socioeconomic factor, and $x_{j}$ is childhood experience factor. $\beta_{i}^{m}$ and $\beta_{m}^{j}$ and are partial effects (i.e., dy/dx_i_, dy/dx_j_) of each variable and evaluated at sample means; $\alpha^{m}$ is the constant term in the regression, ε is the error term. The decomposition of inequality in long-term care need is estimated in Equation (2):

(2) $$\mathrm{CI}=\sum_{i}\left(\beta_{i}^{m}\bar{x_{i}}/\mu \right)C_{i}+\sum_{j}\left(\beta_{j}^{m}\bar{x_{j}}/\mu \right)C_{j}+GC_{\varepsilon }/\mu$$

The $\left(\beta_{i}^{m}\bar{x_{i}}/\mu \right)C_{i}$ denotes the contribution of adult socioeconomic factors, the $\left(\beta_{j}^{m}\bar{x_{j}}/\mu \right)C_{j}$ denotes the contribution of childhood experience factors, and $GC_{\varepsilon }/\mu$ denotes the contribution of ε. Specially, μ is the mean of the dependent variable, $C_{j}$ is the concentration index of $x_{j}$, and $\bar{x_{j}}$ is mean of $x_{j}$. Thus $\beta_{j}^{m}\bar{x_{j}}/\mu$ denotes the elasticity of $x_{j}$. All analyses were performed in Stata 15.0 (Stata Corp LLP, College Station, TX, USA).

## 3. Results

Table 1 shows the definition of variables and summary statistics of independent variables. Overall, 62.46% of respondents lived in the rural area while 37.54% of respondents in the urban area. 22.27% of respondents were registered non-agriculture Hukou. In terms of age distribution, 27.92% of respondents were in the group of age 45–54, 39.67% of respondents were in the group of age 55–64, 24.70% of respondents were in the group of age 65–74, and 7.72% of respondents were in the group of age of 75 and above. Most of the respondents received primary education, accounting for 43.37%, which were followed by those never received formal education, accounting for 25.70%. Only 10.99% of respondents had the chance to be educated in high school and above and 19.94% of the respondents were educated in middle school. It was reported that 10.93% of the respondents were communist party member. What is more, 32.71% of the respondents reported to have consumed alcohol while 35.87% of the respondents reported to have consumed tobacco. Most of the respondents were Han ethics and were covered by basic health insurance in China.

Before estimating inequality in long-term care need among older adults with chronic diseases by using the concentration index directly, we explored the distribution of long-term care need across quantiles of economic status. Table 2 shows the incidence and severity of long-term care need among older adults with chronic disease. ADLs disability and IADLs disability were reported with frequency and percentage, and ADLs score and of IADLs score were reported with the mean and standard deviation across different quantiles of economic status. Overall, 20.44% of respondents were reported with ADLs disability and 19.25% of respondents were reported with IADLs disability. We then displayed the difference in long-term care need across different quantiles of economic status. Especially, 25.08% of respondents in the poorest group (Quantile 1) were reported with ADLs disability, and the percentage declined with the growth of economic status, which reached 16.75% in the richest group (Quantile 5). Meanwhile, 24.89% of respondents in the poorest group (Quantile 1) were reported with IADLs disability, and the percentage declined with the growth of economic status, which reached 15.19% in the richer group (Quantile 4) and 16.43% in the richest group (Quantile 5). The average score of ADLs for the poorest group (Quantile 1) was 0.749, which steadily decreased to 0.462 for the richest group (Quantile 5). It was the same with IADLs score, the highest score, 1.034, was found in the poorest group while the lowest score, 0.548, was estimated in the richer group (Quantile 4) and 0.597 in the richest group (Quantile 5). The concentration index of ADLs disability and of ADLs score among older adults with chronic diseases were −0.085 and −0.109, and the concentration index of IADLs disability and of IADLs score among older adults with chronic diseases was −0.095 and −0.120, all of which were statistically significant, indicating the pro-poor inequalities in long-term care need among older adults with chronic diseases. 

Then we explored what factors and to which extent they influenced the need of long-term care among older adults with chronic diseases in China. Table 3 shows the regression results of long-term care need among older adults with chronic disease in China. Respondents in the middle group were 2.5% less likely to experience physical disability when comparing with the poorest group. Compared with the group of age 45–54, the group of age 55–64, the group of age 65–74, and the group of age 75 and above were significantly associated with 5.4%, 13.6% and 24.0% higher risk of ADLs disability, respectively. Male respondents were estimated to have a 4.2% lower risk of ADLs disability than female respondents. Compared with the illiteracy group, the risk of ADLs disability of the middle school group, and of the high school group and above significantly decreased 4.6%, and 7.4%, respectively. Respondents with party membership were estimated to have a 4.4% lower risk of ADLs disability than respondents without membership. Compared with respondents without experiencing hunger during childhood, respondents experiencing hunger during childhood period 1, period 2, and period 3 had a significant 4.1%, 3.9%, and 5.4% higher risk of ADLs disability, respectively. The risk of ADLs disability of respondents reporting much less healthy significantly increased 5.7% when comparing with respondents reporting much healthier in childhood. 

Second, ADLs score of the middle family consumption group significantly decreased 0.307 unit for the middle group, 0.313 unit for the richer group, and 0.263 for the richest group than the poorest group. Compared with the group of aged 45–54, the group of aged 55–64, the group of aged 65–74, and the group of aged 75 and above were significantly associated with 0.500, 0.828, and 1.266 unit increase in ADLs score, respectively. Urban respondents were estimated to have a 0.233 unit decrease in ADLs score than rural respondents. Using the illiteracy group as reference, the risk of ADLs score of the high school group and above significantly decreased 0.579 unit. Respondents with business health insurance were estimated to have a 0.509 unit decrease in ADLs score than respondents without health insurance coverage. Respondents reporting alcohol consumption were estimated to have a 0.188 unit decrease in ADLs score compared with respondents without reporting alcohol consumption. Respondents with communist party membership were estimated to have a 0.199 unit decrease in IADLs score compared with respondents without membership. Compared with respondents without experiencing hunger during childhood, respondents experiencing hunger during childhood period 1 and period 3 had a significant 0.288 and 0.309 unit increase in ADLs score, respectively. Respondents having vaccine injection under 15 years old were associated with a 0.288 unit decrease in ADLs score than those not. Compared with respondent reporting much healthier in childhood, the ADLs score of respondents reporting average healthy significantly decreased 0.237 unit. 

Third, respondents in the richer group were 4.6% less likely to experience IADLs disability than the poorest group. Compared with the group of age 45–54, the group of age 55–64, the group of age 65–74, and the group of age 75 and above were significantly associated with 4.7%, 12.7% and 25.3% higher risk of IADLs disability, respectively. Male respondents were estimated to have a 2.8% higher risk of IADLs disability than female respondents. Respondents with non-agriculture Hukou were estimated to have a 2.9% lower risk of IADLs disability compared with respondents with agriculture Hukou registration. Compared with the illiteracy group, the risk of IADLs disability of the primary school group, of the middle school group, and of the high school group and above significantly decreased 3.3%, 4.5%, and 8.6%, respectively. Respondents with business health insurance were estimated to have a 7.1% lower risk of IADLs disability than respondents without health insurance coverage. Respondents reporting alcohol consumption were estimated to have a 3.1% lower risk of IADLs disability than respondents without reporting alcohol consumption. Respondents with communist party membership were estimated to have a 3.0% lower risk of IADLs disability compared with respondents without membership. When comparing with respondents without experiencing hunger in childhood, respondents experiencing hunger in childhood period 1, in period 2, and in period 3 had a significant 2.8%, 3.7%, and 5.0% higher risk of IADLs disability, respectively. Respondents having vaccine injection under 15 years old were associated with a 2.9% lower risk of IADLs disability. Respondents having a usual source of care since childhood were associated with a 4.1% lower risk of IADLs disability. Compared with respondent reporting much healthier in childhood, the risk of IADLs disability of respondents reporting much less healthy significantly increased 8.1%. 

Forth, in the last two columns of Table 3, it was estimated that respondents having 0.320 unit decrease of IADLs score for the poorer group, 0.357 unit decrease of IADLs score for the middle group, 0.396 unit decrease of IADLs score for the richer group, and 0.344 unit decrease of IADLs score for the richest group than the poorest group, all of which were statistically significant. Compared with the group of aged 45–54, the group of aged 55–64, the group of aged 65–74, and the group of aged 75 and above were significantly associated with 0.389, 0.876, and 1.492 unit increase in IADLs score, respectively. Education showed comprehensive effects at each stage. IADLs score of the primary groups, of the middle school group, and of the high school group and above significantly decreased 0.383unit, 0.412 unit, and 0.735 unit when comparing with the illiteracy group, respectively. Respondents reporting alcohol consumption were estimated to have a 0.327 unit decrease in IADLs score compared with respondents without reporting alcohol consumption. Respondents experiencing hunger in childhood period 1 and period 3 had a significant 0.218 and 0.325 unit increase in IADLs score than respondents without experiencing hunger during childhood, respectively. Respondents having vaccine injection under 15 years old were associated with a 0.218 unit decrease in ADLs score. Compared with respondent reporting much healthier in childhood, the IADLs score of respondents reporting somewhat healthier significantly decreased 0.122 unit while the IADLs score of respondents reporting much less healthy significantly increased 0.290 unit. 

Based on equation 2, we decomposed inequality in long-term care need among older adults with chronic diseases into its each determinant. Table 4 showed decomposition results. As indicated in equation 2, the contribution of each determinant to the inequality in ADLs disability and IADLs disability was the product of elasticity and the concentration index of each determinant. In this study, we paid a special attention to the contribution of each variable and its percentage of contribution to the total inequality in ADLs disability and IADLs disability. It is the same with ADLs score and IADLs score.

The decomposition results shed light on the relative importance of independent variables contributing to the overall inequality and we focused on variables made the highest contribution. To be detailed, the contribution of the richer group and of the richest group to inequality in ADLs disability accounted for 38.80% and 71.70%. If economic status was equally distributed across economic status groups or if there was no association between economic status and long-term care need, the estimated income-related inequalities would be 38.80% and 71.70 lower, respectively. Each of the other determinants can be interpreted in this pattern. And we found the age group 75 and above and the age group 65–74 took another leading contribution, accounting for 57.71% and 62.72%, respectively. Hukou registration and living in urban areas contributed 8.84% and 38.84% to the overall inequality in ADLs disability, respectively. Receiving middle school education or high school and above education contributed 25.73% and 61.00% to the overall inequality in ADLs disability, respectively. The business health insurance positively contributed to the overall inequality in ADLs disability, accounting for 11.74%. Hunger experience during period 3 in childhood positively contributed 14.73% to the overall inequality in ADLs disability. In the next two columns, the contribution of the richer group and of the richest group to overall inequality in ADLs score accounted for 42.55% and 64.31%, respectively. The age group 75 and above and the age group 65–74 took another leading contribution, accounting for 28.70% and 31.82%, respectively. Hukou registration and living in urban areas contributed 2.44% and 20.11% to the overall inequality in ADLs score, respectively. Receiving middle school education and high school and above education contributed 6.23% and 31.82% to the overall inequality in ADLs score, respectively. The business health insurance positively contributed to the overall inequality in ADLs score, accounting for 10.94%. Hunger experience during period 3 in childhood positively contributed 6.55% to the overall inequality in ADLs score.

As for IADLs disability, the contribution of the richer group and the richest group to inequality in IADLs disability accounted for 86.87% and 56.60%. The age group 75 and above and the age group 65–74 took another leading contribution, accounting for 59.78% and 58.60%, respectively. Hukou registration and living in urban areas contributed 37.52% and 18.33% to the overall inequality in IADLs disability, respectively. Receiving middle school education and high school and above education contributed 24.63% and 73.56% to the overall inequality in IADLs disability, respectively. The business health insurance positively contributed to the overall inequality in IADLs disability, accounting for 23.64%. Hunger experience during period 3 in childhood positively contributed 13.33% to the overall inequality in IADLs disability. 

In the last two columns of Table 4, the contribution of the richer group and the richest group to inequality in IADLs score accounted for 39.63% and 60.86%. The age group 75 and above and the age group 65–74 took another leading contribution, accounting for 21.66% and 25.01%, respectively. Hukou registration and living in urban areas contributed 8.33% and 4.62% to the overall inequality in IADLs score, respectively. Receiving middle school education and high school and above education contributed 11.77% and 29.51% to the overall inequality in IADLs score, respectively. The business health insurance positively contributed to the overall inequality in IADLs score, accounting for 4.93%. Hunger experience during period 3 in childhood positively contributed 4.76% to the overall inequality in IADLs score.

## 4. Discussion

Using two waves of high quality national representative data of CHARLS, the study reveals important findings on income-related inequalities in long-term care need among older adults with chronic diseases in China. First, we find salient presence of long-term care need among older adults with chronic disease in China, an estimation of 20.44% of respondents in need of ADLs care and of 19.25% of respondents in need of IADLs care. Second, substantial pro-poor income-related inequalities in long-term care need are identified among older adults with chronic diseases in China, indicating that low socioeconomic groups had a higher probability of needing long-term care or need more long-term care. Third, decomposition analyses provide important information on the determinants of the inequalities and revealed that family income and education attainment in adulthood, and adverse childhood experience played a significant role in explaining the inequalities in physical disability. We also find different patten of Hukou registration and of living areas. As far as we know, this is the first study to synthesize evidence on the short-term effect of adulthood socioeconomic status and the long-term health effects of individual childhood experience in equalities in long-term care need in China. 

We focus on the specific group of older adults with chronic diseases for their high need of healthcare and of long-term care. The presence of long-term care need among older adults with chronic diseases in China was considerably high. Our study lends support the dose-response effect of aging on long-term care need, namely that the ADLs disability, ADLs score, IADLs disability, and IADLs score was projected to increase significantly as the progress of aging advanced [2]. The results are worrisome. Lacking long-term care insurance coverage, it is possible that the older adults in China with chronic disease tended to overuse healthcare services, which finally driven escalation of healthcare expenditure [14,16]. One study found that the incidence of catastrophic health expenditure for households with chronic disease is at a disconcerting level (34.01%) when comparing with households with non-chronic diseases (13.33%) [13]. Another study found that the incidence of catastrophic health expenditure for households with chronic disease increased from 22.1% in 2008 to 46.9% in 2013 [14]. The real contribution to healthcare expenditure growth due to long-term care need is unclear in this study, which needs further research. However, the number of oldest adults in China with unmet long-term care needs has increased in recent years in a way because of a decline in the availability of family caregivers [5]. As the aging population continues to increase in China, the adverse effect of unmet long-term care need is expected, and it will continually crowded healthcare need and affect the sustainability of health system and insurance in China if appropriate policy responses are not implemented.

We found substantial pro-poor income-related inequalities in long-term care needs, indicating that low socioeconomic groups have a higher probability or more need for long-term care. A better understanding of inequality in long-term care needs should help policy makers gain insights into pathways and strategies [13,14,27], which is necessary for the development of effective policy on universal coverage of long-term care insurance. In this study, we find family consumption and education attainment negatively associated with the increase of long-term care need, and also contributed the most ingredient of the inequalities beyond age factor. First, individuals from lower economic status may underuse necessary health care and other social welfare services due to financial reasons, leading to worsen health status and higher need of long-term care [5,14,16]. Education attainment may also be a proxy for factors beyond family income after controlling for a valid measure [19]. Well-educated adults may possibly access sufficient and high-quality information [19,20,31], which helps them have a better understanding of what to options choose to guarantee healthy conditions. Thus, disadvantages in economic status and in education attainment contribute a lot to the inequalities in long-term care needs, which were consistent with the results of previous studies in developed countries [32,33,34,35]. The results of this study are discouraging for the following reasons when extending universal coverage of long-term care insurance. First, the individuals from low socioeconomic groups usually are not able to pay for formal long-term care serves when they need it. Second, fewer family members are available to be as unpaid informal caregivers considering high opportunity cost when they can find a job. Third, it is possible that they may made suboptimal choice without accessing important information on enrolling appropriate insurance.

We find consistent effects of living in urban areas and having non-agriculture Hukou registration, both of which were negatively associated with a higher need of long-term care. Importantly, Hukou registration and living in urban areas contributed significant gradient to the overall inequality in need of long-term care, supporting the evidence that residents living in urban areas and having non-agriculture Hukou registration have more basic social services and a better health outcome [5,36,37]. However, the contribution pattern of Hukou registration and of living in urban areas is different. Living in urban areas is important for ADLs while Hukou registration for IADLs. It’s reasonable that Hukou registration is inherited from either mother or father at birth, and is always linked with limited job opportunities and lower access to health benefits. Therefore, Hukou registration explained more for instrumental activities of daily living (IADLs) while the residence of living explained more for basic activities of daily living (ADLs), which depends on the accumulative effect of social environment on health. It is also found that male older adults had a significant lower probability of long-term care need than their female counterparts but no significant difference in severity of long-term care need. One plausible explanation is that female adults live 2 years longer than their male counterparts in China and consequently enjoy a longer healthy status duration [6]. However, this advantage can be quickly negated by a significant dose-response effect of aging on physical health. As we find, aging progression exerted the magnificent influence on the probability and severity of long-term care and their income-related inequalities.

This study also gives insights on strategies to address the existing inequalities as early as possible. Exposures and experience in childhood may impact the development of diseases and symptoms in later life [12,35]. Compared with individuals without experiencing hunger in childhood, individuals experiencing hunger in childhood had a significant higher risk of ADLs and IADLs disability and thus result in more need of long-term care, especially the long endurance of hunger experience (Period 3). Hunger experience during period 3 in childhood positively contributed to the overall inequality in ADLs and IADLs difficulty, ranging from 4.76% to 14.73%. Our results also indicated that having vaccinations and access to medical care in childhood positively contributed to the overall inequality in long-term care need. Hence, childhood represents a key life stage for designing interventions to address health inequality in old age. International resolutions, including the Sustainable Development Goals, provide crucial opportunities to address childhood poverty and hunger exposure.

This study has several limitations. First, four indicators—ADLs disability, IADLs disability, ADLs score, and IADLs score—were defined to measure the incidence and severity of long-term care need. However, it should be notified that the severity of long-term care need may vary when considering the importance of each dimension of ADLs/IADLs [5,7]. We did a robust check by defining narrow long-term care need and repeating the results, and it can be further studied in the future. Second, the measures of ADLs and IADLs used in this study were self-reported, which may lead to underestimation of the presence of long-term care need although our subsample of older adults with chronic disease possibly overuse health care. Third, only those with functional disability was screen in our analysis, it is possible that our estimation was underrated due to the omission of those with cognitive impairment. Forth, unobserved premature mortality of adverse childhood experience may bias our estimation of childhood variables to its lower bound. Fifth, not all factors were included in this study such as environmental factors which may also play an important role in determining health in older life. Finally, the study has been careful not to claim a causal relationship between socioeconomic factors and long-term care need based on its cross-sectional design. 

## 5. Conclusions

We found in this study a strong potential need for long-term care and substantial pro-poor income-related inequalities among older adults with chronic diseases in China, indicating that lower socioeconomic groups had a higher probability of needing long-term care or need more long-term care. This study provides important implications of long-term care insurance in China. Considering the high need for long-term care, it is urgent to implement universal long-term care insurance coverage in China, especially for the individuals from lower socioeconomic groups who usually are unable to pay for formal long-term care services when they need them. Several identified groups with severe needs for long-term care in China should be given priority coverage, including the group of lower family income, the group of deficit in education achievement, the group of living in rural areas or having agriculture Hukou registration, and the group experiencing long exposure to hunger during childhood. Due to long-term care insurance coverage, additional gains are expected from less overuse of health care. However, we should be cautious to extend universal coverage of long-term care insurance given the conditions in China. Substantial pro-poor income-related inequalities in long-term care need are identified among older adults with chronic diseases in China. Decomposition analyses revealed that family income and education attainment played a significant role in explaining the inequalities in long-term care need. Hence, getting informal care paid or compensated from job loss is important to induce more family members available to provide care and the government is also responsible to make individuals informed to avoid suboptimal choice before enrolling appropriate insurance, especially from low socioeconomic status. Lastly, the findings suggest that it is critical to begin health interventions to prevent or reduce health inequalities in old age. 

## Figures and Tables

**Table 1:** Variable definition and summary statistics.

Adulthood Factors	Number of People	Percentage	Childhood Factors	Number of People	Percentage
Age group			Hunger experience		
0-Age 45–54	2159	27.9%	0-None of the three periods	2192	28.3%
1-Age 55–64	3068	39.7%	1-Period 1, any one of the three periods	2650	34.3%
2-Age 65–74	1910	24.7%	2-Period 2, any two of the three periods	1265	16.4%
3-Age ≥ 75	597	7.7%	3-Period 3, all of the three periods	1627	21.0%
Gender			Vaccine		
0-Female	4109	53.1%	0-No	1244	16.1%
1-Male	3625	46.9%	1-Yes	6490	83.9%
Marriage			Daily medical source		
0-Married	6804	88.0%	0-No	895	11.6%
1-Divoced	930	12.0%	1-Yes	6839	88.4%
*Hukou*			Childhood health		
0-Agricultural *hukou*	6012	77.7%	0-Much healthier	1173	15.2%
1-Non-agricultural *hukou*	1722	22.3%	1-Somewhat healthier	1428	18.5%
Urban			2-Average	3991	51.6%
0-No	4831	62.5%	3-Less healthy	675	8.7%
1-Yes	2903	37.5%	4-Much less healthy	467	6.0%
Education level					
0-Illiteracy	1988	25.7%			
1-Primary school	3354	43.4%			
2-Middle school	1542	19.9%			
3-High school	850	11.0%			
Insurance coverage					
0-Non-coverage	225	2.9%			
1-Basic health insurance	7150	92.5%			
2-Business health insurance	359	4.6%			
Ethnic group					
0-Minority ethnics	628	8.1%			
1-Han ethnic	7,106	91.9%			
Alcohol consumption					
0-Yes	5204	67.3%			
1-No	2530	32.7%			
Tobacco consumption					
0-No	4960	64.1%			
1-Yes	2774	35.9%			
Party member					
0-No	6889	89.1%			
1-Yes	845	10.9%			
Household size					
0- members 1–2	491	6.4%			
1- members 3–4	4170	53.9%			
2- members 5–6	2099	27.1%			
3- members >=7	974	12.6%			

Note: Dummy variables were generated in regression and 0 denoted reference group. Hunger experience means a time when the family did not have enough food to eat before age 17, and it was reported in three periods, age 0–5, age 6–12, and age 13–17. Vaccine means receiving any vaccinations under 15. Childhood health means health compared to other children of the same age under 15.

**Table 2:** Distribution and the concentration index of long-term care need across quantiles of economic status.

Quantiles of Economic Status	ADL Disability		ADL Score		IADL Disability		IADL Score	
	Frequency	Percentage	Mean	Std. Dev.	Frequency	Percentage	Mean	Std. Dev.
Quantile_1 (Poorest) #	388	25.08	0.749	1.987	385	24.89	1.034	2.354
Quantile_2 (Poorer)	349	22.56	0.644	1.835	317	20.49	0.743	1.999
Quantile_3 (Middle)	301	19.46	0.500	1.477	298	19.26	0.633	1.714
Quantile_4 (Richer)	284	18.36	0.473	1.455	235	15.19	0.548	1.709
Quantile_5 (Richest)	259	16.75	0.462	1.530	254	16.43	0.597	1.828
Overall	1581	20.44	0.566	1.674	1,489	19.25	0.711	1.943
Concentration Index (CI)	−0.085		−0.109		−0.095		−0.120	

Note: # denotes reference group in regression.

**Table 3:** Factors influencing long-term care need among older adults with chronic disease.

Factors	ADLs Disability		ADLs Score		IADLs Disability		IADLs Score	
	dF/dx	Std. Err.	dF/dx	Std. Err.	dF/dx	Std. Err.	dF/dx	Std. Err.
Family consumption								
Quantile_2 (Poorer)	−0.007	0.014	−0.130	0.101	−0.023	0.015	−0.320 ***	0.123
Quantile_3 (Middle)	−0.025 **	0.012	−0.307 ***	0.105	−0.016	0.013	−0.357 ***	0.104
Quantile_4 (Richer)	−0.020	0.022	−0.313 ***	0.108	−0.046 ***	0.016	−0.396 **	0.161
Quantile_5 (Richest)	−0.020	0.012	−0.263 **	0.112	−0.017	0.015	−0.344 ***	0.126
Age group								
55–64	0.054 ***	0.015	0.500 ***	0.089	0.047***	0.013	0.389 ***	0.139
65–74	0.136 ***	0.017	0.828 ***	0.101	0.127***	0.016	0.876 ***	0.140
≥75	0.240 ***	0.036	1.266 ***	0.148	0.253***	0.029	1.492 ***	0.145
Household size								
3–4	−0.002	0.022	0.035	0.158	0.019	0.027	0.244	0.202
5–6	−0.017	0.024	−0.092	0.161	0.026	0.031	0.251	0.202
≥7	0.010	0.026	0.023	0.178	0.012	0.033	0.260	0.187
Male	−0.042 ***	0.014	−0.074	0.092	−0.028 *	0.016	0.094	0.106
Marriage	0.024 *	0.015	0.005	0.117	0.002	0.017	−0.099	0.105
*Hukou*	−0.005	0.017	−0.026	0.107	−0.029*	0.015	−0.121	0.121
Urban	−0.032	0.021	−0.233***	0.087	−0.016	0.016	−0.072	0.116
Education level								
Primary school	−0.012	0.011	−0.084	0.086	−0.033 ***	0.011	−0.383 ***	0.074
Middle school	−0.046 **	0.019	−0.159	0.112	−0.045 **	0.017	−0.412 ***	0.153
High school and above	−0.074 ***	0.021	−0.579 ***	0.146	−0.086 ***	0.014	−0.735 ***	0.106
Insurance coverage								
Basic insurance	−0.021	0.025	−0.150	0.190	−0.035	0.028	−0.049	0.230
Business insurance	−0.036	0.027	−0.509**	0.252	−0.071**	0.024	−0.318	0.278
Ethnic group	0.003	0.015	−0.068	0.135	−0.005	0.034	−0.061	0.199
Alcohol consumption	−0.008	0.005	−0.188 ***	0.044	−0.031 ***	0.006	−0.327 ***	0.053
Tobacco consumption	−0.004	0.016	0.006	0.088	0.007	0.015	0.060	0.092
Party member	−0.044 ***	0.015	−0.199 *	0.118	−0.030 **	0.011	−0.190	0.150
Hunger experience								
Period 1	0.041 **	0.019	0.288 ***	0.088	0.028 **	0.013	0.218 ***	0.104
Period 2	0.039 **	0.020	0.117	0.106	0.037 *	0.020	0.129	0.157
Period 3	0.054 ***	0.021	0.309 ***	0.100	0.050 ***	0.016	0.325 ***	0.119
Vaccine	−0.007	0.012	−0.288 ***	0.090	−0.029 **	0.012	−0.218 **	0.098
Daily medical source	−0.021 *	0.012	−0.030	0.102	−0.041 ***	0.014	−0.124	0.093
Childhood health								
Somewhat healthier	−0.004	0.018	−0.054	0.114	0.006	0.014	0.007	0.105
Average	−0.014	0.011	−0.237 **	0.096	−0.002	0.014	−0.122 ***	0.090
Less healthy	−0.008	0.018	−0.165	0.140	0.025	0.021	0.120	0.141
Much less healthy	0.057 ***	0.022	0.201	0.154	0.081 ***	0.022	0.290 **	0.105

Note: * *p* < 0.1, ** *p* < 0.05, *** *p* < 0.01; dF/dx means partial effects of each variable and evaluated at sample means; Std. Err. means Standard Errors; Province fixed effect was controlled in the regression model and Standardized Error was clustered at the province level.

**Table 4:** Decomposition of inequalities in long-term care need among older adults with chronic disease.

Factors	ADLs Disability		ADLs Score		IADLs Disability		IADLs Score	
	Cont.	Percentage	Cont.	Percentage	Cont.	Percentage	Cont.	Percentage
Family consumption								
Quantile_2 (Poorer)	0.010	−12.20	0.019	−17.00	0.038	−40.59	0.038	−31.37
Quantile_3 (Middle)	−0.001	0.93	−0.001	0.83	−0.001	0.59	−0.001	0.86
Quantile_4 (Richer)	−0.033	38.80	−0.046	42.55	−0.082	86.87	−0.047	39.63
Quantile_5 (Richest)	−0.061	71.70	−0.070	64.31	−0.054	56.60	-0.073	60.86
Age group								
55–64	−0.001	1.04	−0.001	0.75	−0.001	0.91	−0.001	0.67
65–74	−0.053	62.72	−0.035	31.82	−0.056	58.60	−0.030	25.01
≥75	−0.049	57.71	−0.031	28.70	−0.057	59.78	−0.026	21.66
Household size								
3–4	0.000	0.57	0.001	−1.10	0.008	−8.08	0.006	−5.19
5–6	0.000	−0.58	0.000	−0.23	−0.001	0.85	0.000	0.41
≥7	−0.002	2.93	−0.001	0.51	−0.003	3.40	−0.005	4.22
Male	−0.005	6.29	−0.001	0.84	−0.004	4.21	0.001	−0.67
Marriage	−0.010	11.52	0.000	0.20	−0.001	0.76	0.003	−2.32
*Hukou*	−0.007	8.84	−0.003	2.44	−0.036	37.52	−0.010	8.33
Urban	−0.033	38.84	−0.022	20.11	−0.017	18.33	−0.006	4.64
Education level								
Primary school	0.004	−4.46	0.002	−2.15	0.011	−11.41	0.009	−7.92
Middle school	−0.022	25.73	−0.007	6.23	−0.023	24.63	−0.014	11.77
High school and above	−0.052	61.00	−0.035	31.82	−0.070	73.56	−0.035	29.51
Insurance coverage								
Basic insurance	0.004	−4.39	0.003	−2.34	0.006	−6.83	0.001	−0.59
Business insurance	−0.010	11.74	−0.012	10.94	−0.022	23.64	−0.006	4.93
Ethnic group	−0.001	0.71	0.001	−1.06	0.001	−1.13	0.001	−0.70
Alcohol consumption	−0.006	7.29	−0.014	12.92	−0.027	28.25	−0.019	15.51
Tobacco consumption	0.000	0.09	0.000	−0.01	0.000	−0.15	0.000	0.02
Party member	−0.017	20.31	−0.007	6.67	−0.013	13.74	−0.006	4.70
Hunger experience								
Period 1	−0.003	3.27	−0.002	1.71	−0.002	2.20	−0.001	1.20
Period 2	0.000	−0.37	0.000	−0.08	0.000	−0.35	0.000	−0.07
Period 3	−0.012	14.73	−0.007	6.55	−0.013	13.33	−0.006	4.76
Vaccine	−0.003	2.96	−0.010	8.74	−0.011	11.21	−0.006	4.63
Daily medical source	−0.003	4.02	0.000	0.45	−0.007	7.75	−0.002	1.31
Childhood health								
Somewhat healthier	−0.001	0.74	−0.001	0.69	0.001	−0.94	0.000	−0.07
Average	0.002	−2.66	0.003	−3.21	0.000	−0.39	0.001	−1.23
Less healthy	0.000	−0.12	0.000	−0.16%	0.000	0.32	0.000	0.15
Much less healthy	−0.003	3.40	−0.001	0.98%	−0.004	4.66	−0.001	1.02

Note: Cont. denotes the contribution of each independent variable to the inequalities in chronic adult disabilities; Percentage denotes the percentage of each independent variable’s contribution to the inequalities in chronic adult disabilities.
